# Preclinical research platform for uterine leiomyoma: construction optimization and selection of animal models

**DOI:** 10.3389/fvets.2026.1772750

**Published:** 2026-02-23

**Authors:** Dan Wu, Jian Luo, Liyi Lin, Tingting Gu, Zhehui You

**Affiliations:** The Eighth Clinical Medical College of Guangzhou University of Chinese Medicine, Foshan Hospital of Traditional Chinese Medicine, Foshan, China

**Keywords:** animal model, genetically engineered, hormone-induced, uterine leiomyoma, xenograft

## Abstract

Uterine leiomyomas (ULs) are increasingly becoming a disease affecting women’s health and are one of the most common tumors of the female reproductive system. The pathogenesis of ULs remains incompletely understood, and abnormal hormone levels as well as genetic factors are considered to be causative factors. To further investigate the pathogenesis of this disease, explore new treatment options, and validate new therapeutic drugs, reliable animal models are indispensable. The main animal models currently used for studying ULs include four categories: spontaneous animal models, genetically modified animal models, hormone-induced animal models, and xenograft animal models. This paper systematically reviews the advantages and disadvantages of these four major animal models, their applicable scenarios, proposes potential optimization strategies, and organizes a framework for matching research objectives with appropriate animal models, thereby promoting research on the mechanisms and treatment approaches of ULs.

## Introduction

Uterine leiomyomas (ULs) are one of the most common tumors in women, originating from uterine smooth muscle. Epidemiological studies have found that 70% of women will develop this disease before menopause, and one quarter of these patients will have obvious clinical symptoms (1–5). The main clinical manifestations are abnormal uterine bleeding, pelvic pain, infertility, etc (6–10). Surgical operation is currently the main treatment for ULs, but surgery carries risks such as significant trauma, impaired fertility, and disease recurrence (1, 11). There is currently no drug that can shrink ULs and inhibit disease recurrence, so there is an urgent need to develop new treatment methods and drugs, and this relies on reliable animal models (12–14). Reliable animal models are essential platforms for disease pathological mechanism research and evaluation of new therapeutic drugs. Excellent animal models must be able to better simulate the pathological process. Current uterine fibroid animal models cannot fully reproduce the complex pathological process of human ULs, so optimization of current pathological models is needed.

This article conducts methodological comparisons of four main models and proposes some optimization strategies. By analyzing the specific advantages and limitations of various models, a model selection framework is summarized, aiming to provide assistance to researchers.

### Spontaneous animal models

Eker rats (*Rattus norvegicus*), due to their germline mutation in the tuberous sclerosis complex type 2 (Tsc2) tumor suppressor gene, spontaneously develop uterine smooth muscle tumors in approximately 65% of females at 12 to 16 months of age. Because of the convenience of model establishment, this model has become one of the most commonly used models for ULs (15, 16).

Its clear genetic background based on the Tsc2 mutation is closely related to abnormal activation of the mTOR signaling pathway. This rat model exhibits highly similar histomorphology and estrogen/progesterone-dependent manifestations to human ULs (17). Therefore, this model has been widely used in etiological research (18, 19). The model is also extensively applied in drug validation and has been used to evaluate the efficacy of potential drugs such as vitamin D3 and catechol-O-methyltransferase inhibitors (19, 20). However, limitations include long model establishment time, high costs, and the model’s tendency to develop concurrent tumors such as renal and hepatic tumors, which restrict its application (21). In the future, combining Eker rats with hormonal intervention methods may accelerate pathological progression, thereby reducing model establishment time and cost.

Apart from the Eker rat model, Japanese quail and miniature pet pigs (*Sus scrofa*) have also been documented to spontaneously develop ULs. Japanese quail: certain strains (*Coturnix japonica*) can spontaneously develop leiomyomas in the oviduct (22). Their application in ULs research is far less common than the Eker rat model. Studies report that miniature pet pigs (*Sus scrofa*) can also spontaneously develop ULs. This model has the advantage of higher anatomical similarity to the human reproductive system compared to Japanese quail and Eker rats (23). However, high costs and poor animal availability limit the application of this model.

### Exogenous hormone-induced models

Hormone-induced models are created through administration of exogenous estrogen and/or progesterone to animals, mimicking a hyperhormonal state that promotes myometrial hyperplasia and the development of fibroid-like lesions. The methods include single-hormone modeling and combined estrogen-progesterone hormone modeling.

In single estrogen induction models, researchers typically establish models by continuously administering high-dose estrogen. Experimental protocols include intramuscular injection of diethylstilbestrol (40 μg/rat every other day for 9 weeks) or twice-weekly injection of estradiol benzoate (200 μg/rat for 8 weeks). These methods can effectively induce uterine myometrial hyperplasia in rats, accompanied by significantly increased serum estrogen levels and enhanced expression of estrogen and progesterone receptors in myometrial tissues, providing a basic model for subsequent mechanism exploration and pharmacodynamic evaluation (24).

Estrogen and progesterone have synergistic effects in the occurrence and development of fibroids, making combined induction a major modeling strategy. According to the timing of hormone administration, protocols can be divided into sequential induction and concurrent induction. Short-term sequential induction protocols (4–5 weeks) are based on daily injection of estradiol benzoate, with progesterone added at the final stage; long-term protocols (≥12 weeks) involve 8–12 weeks of estrogen monotherapy induction followed by several weeks of progesterone supplementation, more closely mimicking the natural progression of human fibroids. Concurrent induction administers both hormones throughout the entire cycle and may have advantages in promoting hormone receptor expression. These protocols together construct a research spectrum ranging from rapid screening to chronic progression simulation (24–26).

To build animal models better suited for “disease-syndrome combination” research in integrated Chinese and Western medicine, researchers have developed multi-factor composite stimulation models. Based on conventional combined estrogen-progesterone induction, this model integrates chronic unpredictable stress stimulation and epinephrine intervention, successfully reproducing the core characteristics of the key Chinese medicine syndrome “Qi Stagnation and Blood Stasis” while simulating Western pathological changes of ULs. Experimental data show that compared to single-hormone induction models, this composite model not only stably presents typical pathophysiological changes of ULs but also demonstrates better model stability and reproducibility, providing a research platform with greater physiological and pathological relevance for systematically exploring synergistic mechanisms between Chinese and Western medicine and evaluating compound drug efficacy (27).

Although hormone-induced models can effectively simulate the hormone-driven process of ULs, they have significant limitations. The success rate of single estrogen induction is relatively low, and it typically induces only diffuse myometrial hyperplasia, making it difficult to form clinically typical isolated fibroid nodules, so its application is mainly limited to scenarios specifically studying the isolated effects of estrogen. Combined estrogen-progesterone induction improves model stability through sequential or concurrent dosing strategies, constructing a research spectrum from rapid screening to chronic progression simulation, but standardized protocols are still lacking.

Currently, key parameters including hormone type selection and administration protocols (dosage, frequency, duration) are not unified, affecting the reliability and comparability of research results. Future efforts need to establish standardized operating procedures and systematically evaluate model similarity to human diseases using multi-omics technologies to improve the predictive value of models and promote the effective translation of preclinical discoveries into clinical treatments.

### Xenograft models

Xenograft models primarily consist of tissue implantation and cell transplantation. Tissue implantation involves grafting human-derived uterine leiomyoma tissue into immunodeficient mice (e.g., SCID mice) subcutaneously or beneath the renal capsule, often supplemented with sustained hormone-release devices to preserve the histological characteristics of the original ULs (25, 28, 29). Advances in methodology have progressively improved the historically low efficiency of cell transplantation, enabling its successful application in pharmacodynamic evaluations of compounds such as resveratrol (30, 31). These models closely replicate the histopathological morphology of human ULs. However, their broader application remains limited by technical complexity, stringent experimental requirements, and high costs. Future directions include developing more cost-effective host systems and establishing standardized protocols to reduce modeling difficulty and expense.

### Genetically modified animal models

Genetically engineered models are established through specific editing of key pathogenic genes, enabling them to simulate specific genetic alterations found in human ULs. These models can be used for in-depth analysis of UL pathogenesis and for validating targeted therapies. The Tsc2 knockout model utilizes the Cre-loxP system to achieve uterine-specific gene deletion, thereby inducing ULs. The PR-Cre-driven model can form ULs with pathological features similar to those in humans within 24 weeks, while the Amhr2-Cre model primarily induces myometrial hyperplasia for modeling purposes (17, 32). The MED12 mutation model introduces the human-derived c.131G > A mutation, which not only promotes UL formation but also results in a higher tumor incidence (up to 80%) and earlier disease onset when the endogenous Med12 gene is knocked out (33, 34). High technical complexity and cost are the main challenges currently faced by genetically engineered models. However, they hold unique value for research into the influence of genetic background on ULs. At the same time, modification of a single gene appears insufficient to fully replicate the complex pathological process of human ULs. In the future, developing composite models with multiplex gene editing could better simulate the complex genetic background of the disease (see Tables 1, 2).

**Table 1:** Overview of UL animal models: a comparative guide.

Model	Methodology	Primary advantages	Primary limitations	Optimal research applications
Spontaneous Model	Spontaneous tumorigenesis due to genetic mutation (e.g., Tsc2 in Eker rat)	High pathological similarity to humans; No artificial intervention required; Ideal for etiological studies.	Long latency period; High cost; Concurrent development of other tumors (e.g., renal).	Gene–environment interactions; Pathogenesis studies; Chemoprevention research.
Hormone-Induced Model	Administration of exogenous estrogen (E2) and/or progesterone (P4)	Low cost; Simple operation; Controllable timeline; Suitable for large-scale studies.	Typically induces diffuse hyperplasia rather than discrete nodules; Lack of standardized protocols.	Preliminary drug efficacy screening (especially for hormonal therapies); Pathophysiological studies.
Xenograft Model	Transplantation of human-derived tissue/cells into immunodeficient mice	Preserves human tumor heterogeneity and microenvironment; Highest translational value.	Dependent on expensive immunodeficient animals; High cost; Inter-donor sample variability.	Preclinical precision efficacy/toxicity evaluation; Personalized therapy research.
Genetically Engineered Model	Targeted gene editing (e.g., Tsc2, MED12)	Enables definitive mechanistic studies with clear causality; Ideal for target validation.	Technically complex; Very high cost; Long generation time.	Validation of specific gene/pathway function; Mechanistic studies of targeted drug action.

**Table 2:** Key optimization parameters for UL animal models.

Model	Key parameters for optimization	Primary optimization goals
Spontaneous Model	Induction timeline control Tumor burden management	Reduce latency, minimize incidental tumors, enhance phenotype specificity
Hormone-Induced Model	Standardization of hormone type/dose/regimen Animal genetic background selection	Improve reproducibility, induce discrete nodules, better mimic human disease progression
Xenograft Model	Host system cost & accessibility Tissue/cell preparation & engraftment protocols Donor tissue characterization	Reduce cost and technical barriers, account for tumor heterogeneity, improve engraftment rate
Genetically Engineered Model	Multi-gene editing strategies	Better simulate complex genetics, achieve spatiotemporal control, increase translational relevance

## Discussion

The development of animal models for ULs has advanced beyond simple hormone induction techniques, progressively establishing a comprehensive research system that incorporates genetic engineering, xenograft transplantation, and other technologies. Different types of models play distinct roles in disease research based on their technical characteristics: spontaneous models can directly demonstrate the interaction between genetic background and environmental factors, providing a unique perspective for analyzing the complex etiology of the disease; hormone-induced models are simple to operate and cost-controllable, making them widely used in preliminary pharmacological screening of candidate drugs; xenograft models can best simulate the pathological features of human ULs and serve as a key bridge between basic research and clinical translation; genetically engineered models enable precise regulation of key pathogenic genes and are ideal tools for target validation and molecular mechanism studies.

From a comparative pathology perspective, the limited success in translating findings from existing animal models into clinical therapies can be attributed to several key factors. Firstly, there are significant species-specific differences in reproductive endocrinology and uterine anatomy between common model organisms (e.g., rodents) and humans, which limit the fidelity of the hormone-responsive tumor microenvironment. Secondly, most models fail to recapitulate the full heterogeneity of human ULs in terms of size, number, location, and molecular subtypes, reducing their predictive value for drug testing. Finally, many current approaches overlook critical non-cell-autonomous factors such as immune system interactions and extracellular matrix remodeling, which are now recognized as crucial in UL pathogenesis. These deficiencies highlight that current modeling approaches remain insufficient for fully simulating the complex, multi-factorial nature of the human disease.

Current research on UL animal models still suffers from insufficient standardization. Particularly in hormone-induced models, the selection of animal strains, hormone intervention doses, and efficacy evaluation metrics have not yet been unified, limiting the comparability of results across different studies. Given that a single model can hardly fully recapitulate the pathophysiological process of human ULs, future research should move beyond the limitations of individual models and adopt integrated strategies employing multiple models. For example, genetically engineered models can be used to validate target mechanisms, while xenograft models can be applied for preclinical efficacy assessment, thereby forming a complete research chain from basic mechanisms to clinical translation. This approach fully leverages the strengths of various models while significantly improving research efficiency and translational value.

By continuously advancing technological innovation, strengthening standardization, and promoting interdisciplinary collaboration, research on UL animal models will undoubtedly provide a more powerful platform for exploring disease mechanisms and developing novel therapeutic approaches.

## Conclusion

In summary, multiple animal models—including spontaneous, hormone-induced, xenograft, and genetically engineered systems—provide complementary advantages for uterine leiomyoma (ULs) research. Current challenges primarily involve insufficient standardization and the limited representation offered by individual models. Future efforts should focus on integrated multi-model strategies and the optimization of experimental protocols to better simulate human disease and accelerate the development of therapeutic approaches.

## Data Availability

The original contributions presented in the study are included in the article/supplementary material, further inquiries can be directed to the corresponding author.
